# Evaluation of VP4-VP2 sequencing for molecular typing of human enteroviruses

**DOI:** 10.1371/journal.pone.0311806

**Published:** 2024-12-10

**Authors:** Kouichi Kitamura, Minetaro Arita

**Affiliations:** Department of Virology II, National Institute of Infectious Diseases, Musashi-murayama, Tokyo, Japan; Taif University, SAUDI ARABIA

## Abstract

Enteroviruses and rhinoviruses are highly diverse, with over 300 identified types. Reverse transcription-polymerase chain reaction (RT-PCR) assays targeting their VP1, VP4, and partial VP2 (VP4-pVP2) genomic regions are used for detection and identification. The VP4-pVP2 region is particularly sensitive to RT-PCR detection, making it efficient for clinical specimen analysis. However, a standard type identification method using this region is lacking. This study aimed to establish such a method by examining the divergence of VP4-pVP2 amino acid sequences between enterovirus and rhinovirus prototypes. Pairwise analysis of 249 types indicated a 95% threshold for enterovirus intra-species identification but not for rhinovirus prototypes. Protein BLAST search analyses of representative enterovirus prototypes, including EV-A71, EV-D68, CVA6, CVA10, CVA16, and polioviruses (PVs), validated the 95% threshold for typing, with a few exceptions such as PV1-PV2 and CVA6-CVA10, as well as some EV-C types. This study proposes a criterion for typing based on VP4-pVP2 amino acids, which can aid in rapid enterovirus diagnosis during routine clinical or environmental surveillance and emergency outbreaks. Our research confirms the reliability of the suggested VP4-pVP2-based threshold for typing and its potential application in laboratory settings.

## Introduction

In the genus *Enterovirus* under the family *Picornaviridae*, human enteroviruses (EVs) are classified under four species (*Enterovirus alphacoxsackie*, *E*. *betacoxsackie*, *E*. *coxsackiepol*, and *E*. *deconjuncti* formerly *Enterovirus A—Enterovirus D*) and human rhinoviruses (RVs) under three species (*E*. *alpharhino*, *E*. *betarhino*, and *E*. *cerhino* formerly *Rhinovirus A—Rhinovirus C*) (https://ictv.global/report/chapter/picornaviridae/picornaviridae/enterovirus). The single-stranded RNA genomes of EVs and RVs contain a single open reading frame that encodes a polyprotein comprising four structural proteins (VP1–VP4) and seven non-structural proteins (2A–2C and 3A–3D). To date, more than 300 types of human EVs and RVs have been reported [1]. In particular, polioviruses (PVs), classified as *E*. *coxsackiepol*, are well-known poliomyelitis pathogens. Despite global efforts to eradicate them, PVs remain a public health concern [2]. Non-polio enteroviruses (NPEVs) include coxsackieviruses (CVs), echoviruses (Es), and notable EVs, such as EV-A71 and EV-D68. Although most EV infections are asymptomatic, EVs and RVs are associated with various clinical manifestations, such as respiratory, gastrointestinal, and cutaneous symptoms, and occasionally cause severe diseases of the central nervous system or the myocardium [1]. Some EVs are rarely associated with acute flaccid paralysis (AFP), including poliomyelitis and acute flaccid myelitis (AFM) [3]. EV-A71 outbreaks typically affect infants and young children. In severe cases, complications such as encephalitis and meningitis may develop, occasionally resulting in mortality. EV-A71 outbreaks have been frequently reported in the Asia-Pacific region in the past and have been recently reported in Europe [4, 5]. EV-D68 has been identified worldwide, with a major outbreak reported in North America and Europe, affecting a large number of individuals, particularly those with respiratory and neurological diseases, including AFM [3, 5]. Clinical and environmental surveillance is necessary to monitor NPEVs that cause severe symptoms and PVs on the brink of eradication.

Virus isolation in cell culture is the traditional method for the identification of EVs and RVs. Numerous serotypes have been identified via serum cross-neutralization assays. However, since the neutralization assay is time-consuming and requires a considerable number of antisera specific to each distinct serotype, molecular typing based on the capsid-coding region is now routinely used to identify new types rather than serotypes. Various reverse transcription-polymerase chain reaction (RT-PCR) methods targeting different regions of the viral genome have been developed for the direct identification of EV and RV types from isolates or clinical specimens [6–11]. The VP1 region, where the major neutralization determinants are located, is highly divergent among types and is associated with serotyping using the neutralization assay [6, 12]. Therefore, VP1 sequencing is considered the gold standard for molecular typing and is recommended by the World Health Organization (WHO) enterovirus surveillance guidelines [13, 14]. The thresholds of 75% nucleotide and 88% amino acid sequence identity with the prototype are used for typing [12, 15]. Currently, Enterovirus Genotyping Tool Version 0.1 (National Institute of Public Health and the Environment the Netherlands, RIVM; https://www.rivm.nl/mpf/typingtool/enterovirus/), which is based on these thresholds, is widely used for typing [16]. A partial VP1 sequence can be used for typing, whereas the complete VP1 sequence (∼900 nucleotides) is required for the assignment of new types without serological confirmation [13, 14].

Complete VP4 and partial VP2 (VP4-pVP2) regions serve as alternative targets for identifying EVs and RVs. Typically, the VP4 region is highly conserved, in contrast to the relatively divergent VP2. A previous study indicated a correlation between EV typing based on the VP2 sequence and that based on the VP1 sequence, albeit with a limited number of analyzed EV serotypes [17]. The VP4-pVP2 semi-nested RT-PCR, which employs a relatively short amplicon and highly conserved primer sequences (EVP2, EVP4, and OL68-1), exhibits higher detection sensitivity for many EV and RV types compared with VP1 RT-PCR [8]. Specifically, VP4-pVP2 is commonly used for RV identification owing to the challenges associated with RV detection using VP1 RT-PCR. In instances wherein VP1 sequencing proves unsuccessful in EV typing, the use of the VP4-pVP2 region is recommended as an alternative [14]. However, unlike VP1 typing, no standardized method for typing using the VP4-pVP2 sequence exists. The Basic Local Alignment Search Tool (BLAST) [18] is commonly used for type identification. There is a need for the establishment of a consensus on a standardized typing method using VP4-pVP2 sequencing. Therefore, this study aimed to investigate the divergence of the VP4-pVP2 amino acid sequence between EV and RV prototypes. We analyzed the pairwise identities of 249 prototype sequences to identify the threshold for VP4-pVP2 typing. Additionally, we performed a BLAST search using the selected prototype sequences to verify whether the proposed threshold could be applied to type identification.

## Materials and methods

### Virus sequences

The prototype genome sequences of EVs (*E*. *alphacoxsackie*, *E*. *betacoxsackie*, *E*. *coxsackiepol*, and *E*. *deconjuncti*) and RVs (*E*. *alpharhino*, *E*. *betarhino*, and *E*. *cerhino*) were obtained from GenBank using the National Center for Biotechnology Information (NCBI) Entrez retrieval system according to the accession numbers listed by the International Committee on Taxonomy of Viruses (ICTV, https://ictv.global/report/chapter/picornaviridae/picornaviridae/enterovirus) as of November 2022. Prototypes registered with partial sequences not containing the VP4-pVP2 region were excluded from the analyses. PV Sabin strains were also excluded as they are 99–100% identical to the corresponding wild-type PVs with the VP4-pVP2 amino acid sequence. CVA18 strain G13 (accession number: AF499640) in the ICTV list, which is now reclassified as CVA13 [19], was omitted from this study because the CVA13 prototype already contains the AF499637 strain. Finally, a total of 249 prototypes were included in the analysis. A multi-FASTA file containing 249 deduced amino acid sequences corresponding to the VP4-pVP2 amplicon, except reverse primer sequences, was generated and aligned using Geneious Prime software version 2023 (Dotmatics, Boston, MA, USA).

### Prototype sequence analysis

The VP4-pVP2 amino acid sequences (corresponding to VP4 1–69 aa and VP2 1–76 aa in PV1, accession number: V01149) of the 249 prototypes were aligned using the MUSCLE algorithm in MEGA software (version 11) [20]. Phylogenetic analysis was performed using the Saitou and Nei neighbor-joining algorithms in MEGA 11 [21]. Furthermore, pairwise identity comparison with gap was performed using the GENETYX-Mac software (version 20; GENETYX, Tokyo, Japan). A heatmap and histograms of the identity percentages were created using GraphPad Prism software (Dotmatics, Boston, MA, USA).

### Blastp analysis

The VP4-pVP2 amino acid sequences of the selected prototypes were used as queries for the Protein BLAST search (blastp, version 2.13.0+) of the NCBI BLAST tool (https://blast.ncbi.nlm.nih.gov). Query sequences were applied to the reference database of “non-redundant protein sequences (nr)” (update date:2023/01/12). Default values were used for the algorithm parameters (matrix, BLOSUM62; gap existence cost, 11; and gap extension cost, 1). From the results obtained for the top 100 hit references, the information on the assigned type (“Scientific Name”) and identity score (“Per. ident") was obtained for each prototype. Reference sequences that could only be assigned at the genus or species levels or were mislabeled (such as “*Salmonella*” or “*Campylobacter*”) were excluded from the results. The potentially mislabeled strains ADP20664 (PV2), AJO68285 (E9), ACX31093 (CVA9), BAD01622 (CVA9), AWD73908 (CVA9), and ACX31095 (CVA18) were also excluded. Types with spelling variants were summarized.

## Results

### Pairwise identity comparison of the VP4-pVP2 amino acid sequences of EV and RV prototypes

**Fig 1:**
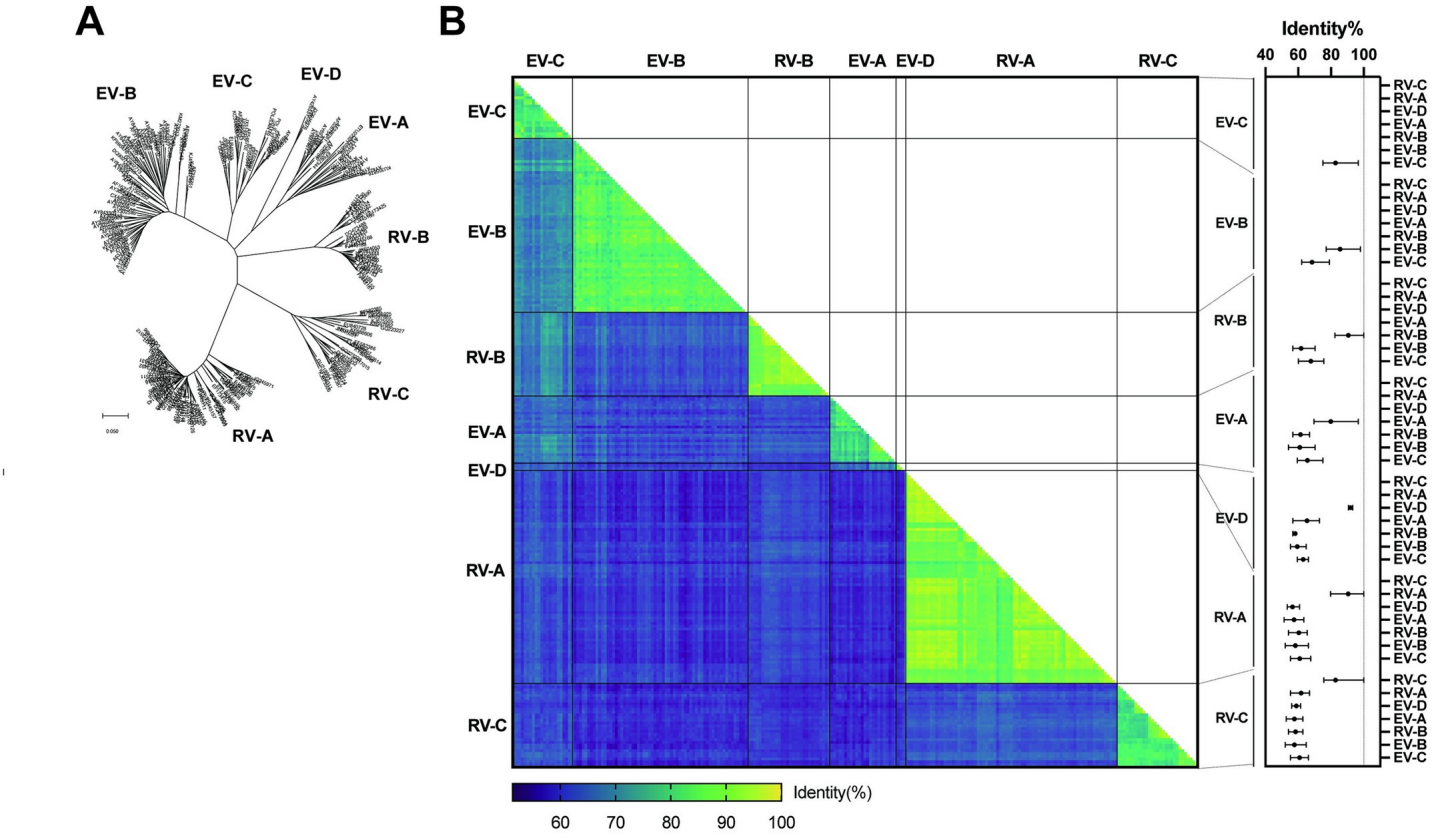
Relationship between 249 EV and RV prototypes based on the VP4-pVP2 amino acid sequence. The assigned species of *Enterovirus alphacoxsackie*, *E*. *betacoxsackie*, *E*. *coxsackiepol*, *E*. *deconjuncti*, *E*. *alpharhino*, *E*. *betarhino*, and *E*. *cerhino* prototypes are labeled EV-A, -B, -C, -D, RV-A, -B, and -C, respectively. (**A**) Phylogenetic analysis. Sequences were aligned using the MUSCLE algorithm. The phylogenetic tree was constructed using the neighbor-joining method with Poisson correction. (**B**) (left) Matrix of pairwise identity comparison among 249 prototypes. Identity percent is indicated using color-coded boxes; (right) Mean, min, and max values of identity percent for inter- and intra-species comparisons.

From the 249 prototype genome sequences (24 *E*. *alphacoxsackie*, 63 *E*. *betacoxsackie*, 22 *E*. *coxsackiepol*, 3 *E*. *deconjuncti*, 77 *E*. *alpharhino*, 30 *E*. *betarhino*, and 30 *E*. *cerhino*), the deduced VP4-pVP2 amino acid sequences were used for phylogenetic analysis (Fig 1A). All the prototypes showed a clear distinction, with seven clusters assigned to the species. Furthermore, the results of pairwise identity comparisons using the VP4-pVP2 amino acid sequences of the 249 prototypes are shown in Fig 1B. The identity values between prototypes within species tended to be high, whereas interspecies-based values tended to be low (Fig 1B). Low interspecies identity values corresponded to the distances between clusters in the phylogenetic tree. The basic statistics of the pairwise identities are summarized in Fig 1B (right panel) and S2 Table.

**Fig 2:**
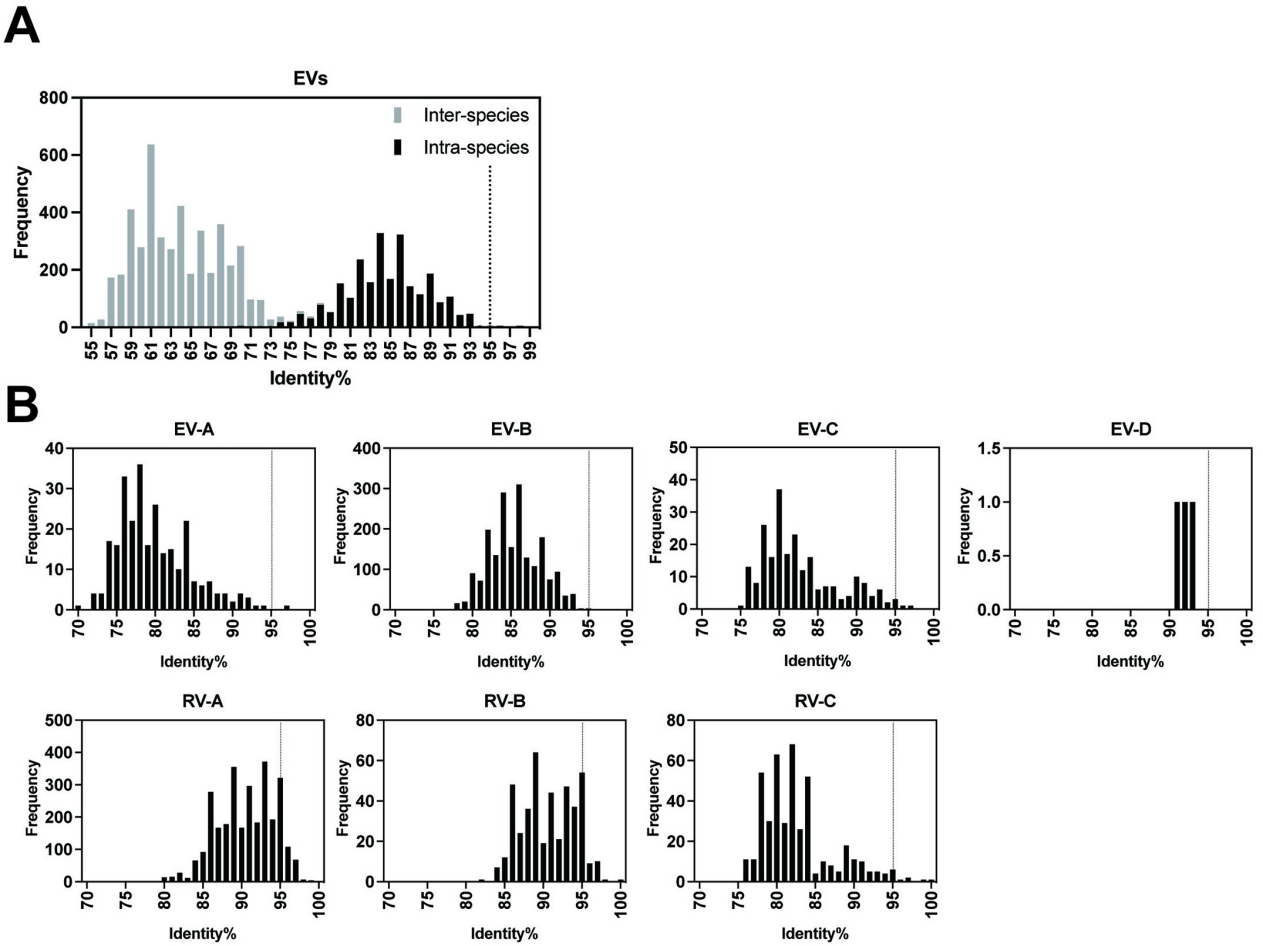
Histogram of pairwise identity frequency based on the VP4-pVP2 amino acid sequence. (**A**) Comparisons among total EV prototypes. (**B**) Intra-species comparisons for EVs and RVs are separately presented (EV-A, -B, -C, -D, RV-A, -B, and -C). The proposed 95% threshold is indicated as a dotted line. EV pairs showing >95% identity are listed in Table 1.

The maximum intra-species identities for some RV species were 100%, indicating identical amino acid sequences in the VP4-pVP2 regions among some RV prototypes. The frequency of the pairwise identity values of the EVs is shown as a histogram (Fig 2A). The identity values were predominantly distributed as bimodal, namely, inter-species and intra-species identities with an overlap of 70%–79%. In the histogram of intra-species identities, the peak was at bin 95% with five exceptions. Histograms of pairwise identities within species for EVs and RVs are presented in Fig 2B. Among the EVs, one *E*. *alphacoxsackie* pair, one *E*. *betacoxsackie* pair, and three *E*. *coxsackiepol* pairs showed >95% identity (Table 1), whereas several RV pairs, especially *E*. *alpharhino* and *E*. *betarhino*, showed >95% identity. These data suggest the use of 95% identity as the threshold for EV intra-species discrimination.

**Table 1:** EV pairs with >95% identity.

Species	Pair	Identity%	blastp result
*E*. *alphacoxsackie*	EV-A91-EV-A121	96.62	-
*E*. *betacoxsackie*	EV-B113-EV-B114	97.97	-
*E*. *coxsackiepol*	PV1-PV2	95.27	Separated
*E*. *coxsackiepol*	CVA20-EV-C102	96.62	Closely related
*E*. *coxsackiepol*	EV-C105-EV-C109	95.94	Separated

### Blast search analyses using the VP4-pVP2 sequences of EV prototypes

**Fig 3:**
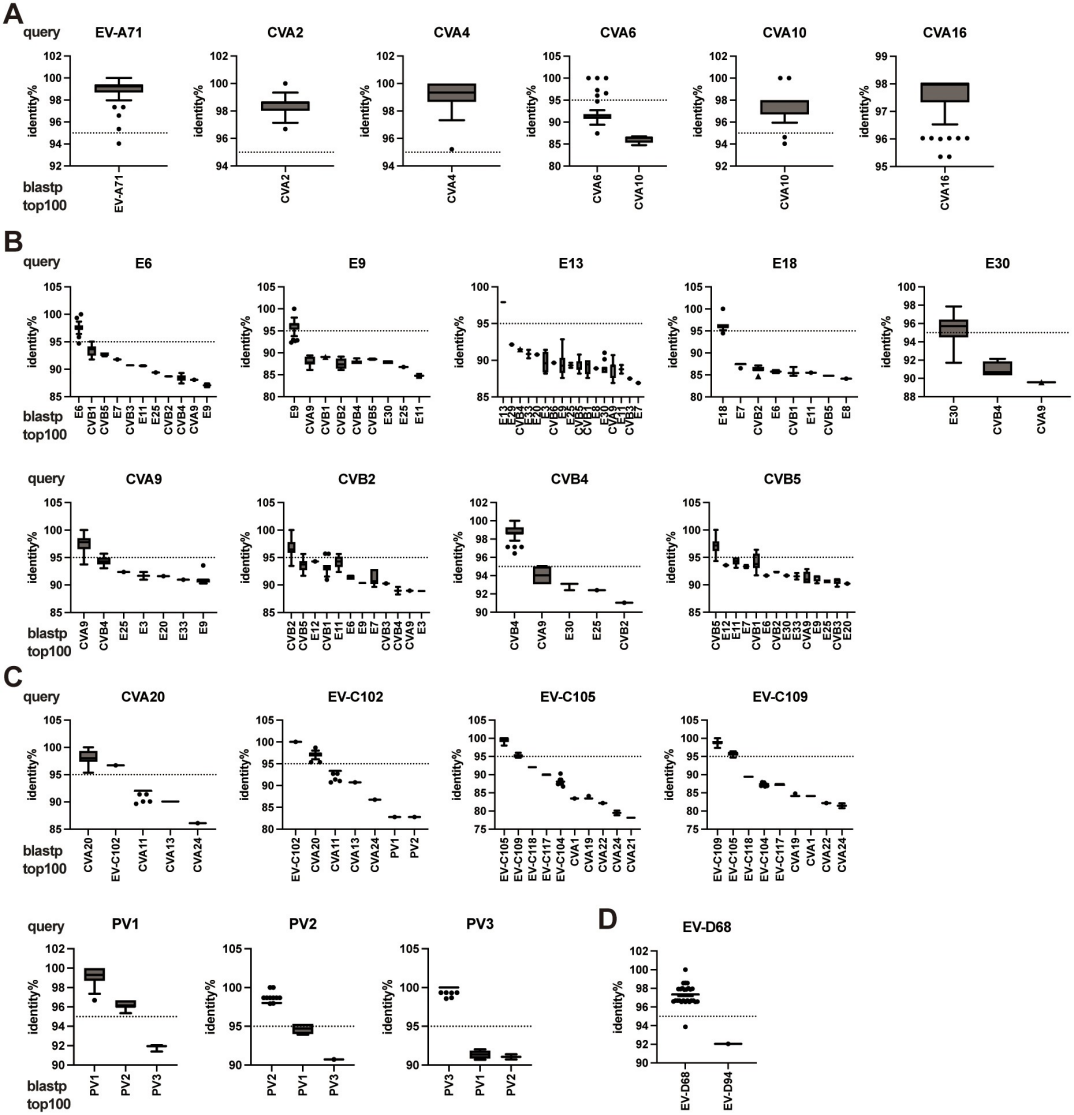
Distribution of identities between prototype and matched strains. Box plots represent the identity percent between the indicated prototypes as a query in the blastp analysis and the top 100 matched references with assigned types. (**A**) *E*. *alphacoxsackie*, (**B**) *E*. *betacoxsackie*, (**C**) *E*. *coxsackiepol*, and (**D**) *E*. *deconjuncti*. Untyped or mislabeled references were excluded from the blastp analysis results.

To confirm the existence of a 95% threshold in intra-type pairwise identities, the VP4-pVP2 sequences of the selected prototypes were subjected to a BLAST search via blastp analysis using the nr database. As the major EV types that cause hand-foot-mouth-disease (HFMD), the queries for EV-A71, CVA2, CVA4, CVA10, and CVA16 matched only the corresponding reference types from the top 100 results (Fig 3A). Among them, only one isolate in EV-A71 (94.04%) and two isolates in CVA10 (94.63 and 94.04%) showed an identity below 95%. The result of CVA6, which is also a major causative agent of HFMD, showed 91.67% identity on average with the matched CVA6 isolates (Fig 3A). Furthermore, the results for *E*. *betacoxsackie* species (E6, E9, E13, E18, E30, CVA9, CVB2, CVB4, and CVB5) showed the corresponding type with >95% identity besides many other types under the threshold (Fig 3B). For PVs, three types were discriminated from each other, although the identities of their PV1-PV2 pairwise comparisons were higher than 95% (PV1 prototype to PV2 references: 95.36%–96.69%) (Fig 3C), as expected based on the prototype comparison (Table 1). Expectedly, CVA20-EV-C102 and EV-C105-EV-C109 comparisons had >95% identities (Fig 3C). The identity between CVA20 prototype and sole EV-C102 reference was 96.69%, and that between EV-C102 prototype and CVA20 references was 95.36%–98.68%. Similarly, EV-C105 prototype and EV-C109 references showed 94.66%–96.03% identities, and EV-C109 prototype and EV-C105 references showed 94.70%–96.43% identities (Fig 3C), consistent with the prototype comparisons (Table 1). Considering only one strain was registered as EV-C102 in the database, its identity within the EV-C102 type could not be determined. The results for EV-D68 showed only one different type, EV-D94, with 92.05% identity (Fig 3D). The results indicate that a 95% threshold exists between inter- and intra-type pairwise identities, with a few exceptions.

## Discussion

During pathogen surveillance, samples from patients with clinical manifestations, including AFP, HFMD, herpangina, meningitis, or respiratory diseases, are generally subjected to EV testing. EV molecular typing has been established based on the nucleotide/amino acid sequence of the entire or partial VP1 region of EV genomes since this region is highly diverse among serotypes [6, 12]. Species-specific VP1 primers are also used in national laboratories, enhancing the sensitivity of detection [7, 10]. However, the efficacy of RT-PCR targeting the VP1 region is not generally high owing to the absence of conserved nucleotide sequences flanking the VP1 region or within it [9]. Although some sensitive RT-PCR assays targeting the entire capsid region of EVs have been developed, methods for nucleotide sequencing of the VP1 region that are applicable in routine surveillance have not been established [11, 22]. In EV surveillance, RT-PCR assay targeting the VP4-pVP2 region is also used owing to its simplicity as well as high sensitivity and specificity [8]. Despite the usefulness of the VP4-pVP2 region for detection, its utility and limitations in identifying EVs remain to be established [17].

Several RVs have pairwise identity values greater than 95%, with some having a value of 100% (Fig 2B). For RVs, the 13% distance threshold for the VP1 nucleotide sequence has been proposed for typing, while the amino acid sequence is not used [23]. Even in neutralization assays, certain sets of RV serotypes show cross-reactivity with antisera [24]. Using the VP4-pVP2 amino acid sequence for RV typing is challenging; however, it can be used for the classification of RV species A, B, or C. In the case of EVs, the threshold for intra-species identity can be set to 95% based on the histogram of EV pairwise identities (Fig 2A), except for five pairs (Table 1). PVs are generally identified using VP1 RT-PCR with either pan-PV or type-specific primers but can also be detected through VP4-pVP2 RT-PCR with other EVs [25]. EV-A91 and -A121 have emerged, alongside EV-A76, -A89, and -A90 [26, 27]. The pair with the highest identity was EV-B113 and EV-B114 (97.97%, three amino acids different), which are known as non-human EVs [28]. The close similarity of the structural region between CVA20–EV-C102 and EV-C96–EV-C99 has been previously demonstrated [15].

In this study, we did not include intra-type comparison with non-prototypes in the distribution analysis owing to sampling bias among types, which made it inappropriate to include a comprehensive prototype analysis. Instead, as a practical strategy for VP4-pVP2 typing, a BLAST search was performed separately for the selected EVs. Using this analysis, matched sequences registered in the NCBI protein database were obtained and compared for each prototype. The results demonstrated that the proposed 95% threshold could be used to accurately identify EV types from the top 100 hit sequences in most of the tested prototypes, such as PVs and the major pathogens of HFMD (EV-A71, CVA2, CVA4, CVA10, and CVA16), meningitis (CVA9, CVB2, CVB4, CVB5, and E6, E9, E13, E18, and E30), and AFP (EV-D68).

However, a few exceptions to the 95% threshold in the BLAST search results were identified. In the analysis with the CVA6 prototype as query, most of the matched CVA6 references clustered at approximately 92% identity, indicating a relatively high divergence of major strains from the prototype. Among the four CVA6 clades (A–D), the prototype of the Gdula strain was the sole member of clade A whereas the predominant strains belonged to clade D [29]. However, this aspect may not affect the relevance of the BLAST search results for CVA6 identification because a large number of CVA6 strains in the database still showed higher identities than other types. The CVA20 and EV-C102 pairs were also exceptions, as mentioned above. When the CVA20 prototype was used as a query, the inter-type identities of matched CAV20 strains were high (median: 98.01%, interquartile range: 97.35%–99.34%); however, the lowest value was 95.36%, and the identity with EV-C102 was 96.69% (Fig 3). The pairs EV-A91–EV-A121 and EV-B113–EV-B114, which has >95% identity based on prototype comparisons, were not analyzed using BLAST search because only prototypes and one additional EV-A91 strain that was a 100% match to the prototype VP4-pVP2 were registered. Recently, EV-C105 and EV-C109 were found to be associated with respiratory diseases [30]. From the nr database, 13 EV-C105 strains and 7 EV-C109 strains were obtained, and the maximum inter-type identity reached 96.03% but was still discriminable to EV-C105 intra-type identities (100%–98.01%) and vice versa (Fig 3C).

Some currently circulating EV strains might be significantly divergent from old prototypes. This study determined the distance between prototypes or between prototype and registered strains in the nr database. As mentioned above, we did not compare between non-prototype strains and we did not use non-prototypes as query for the BLAST search. Due to these limitations, the BLAST search results require careful interpretation. Alternatively, a multi-FASTA file containing the VP4-pVP2 amino acid sequences of the 249 prototypes (S1 Data) can be used as an in-house database for type identification.

To maintain a polio-free status within the framework of the Global Polio Eradication Initiative, various countries have implemented clinical and environmental surveillance measures [31]. Recently, a novel oral polio vaccine type 2 (nOPV2) strain has been developed (accession number: MZ245455) [32]. In regions where this novel live vaccine is deployed, there is a potential for widespread detection through these surveillance measures. nOPV2 is modified from Sabin 2, but the primer sites and amino acid sequence of the VP4-pVP2 region are not changed (EVP2: nt 505–529, EVP4: nt 602–621, OL68-1: nt 1244–1263, amino acid sequence: nt 809–1243). NPEVs are frequently identified through surveillance efforts [33–37]. Environmental samples may contain mixtures of EVs. The application of high-throughput EV identification using next-generation sequencing with VP4-pVP2-based typing may be a valuable tool for environmental surveillance. Although VP1-based typing is still the gold standard as per the WHO-recommended guidelines, the VP4-pVP2-based method can be used as a supplemental method should the VP1 PCR not work. In addition to regular surveillance, rapid and sensitive detection using the VP4-pVP2 RT-PCR assay and type identification may be beneficial in emergency response situations.

## Conclusion

A 95% threshold of pairwise identity for the VP4-pVP2 amino acid sequence of EVs exists between intra-species and intra-type comparisons, with a few exceptions. More practically, our results indicate that BLAST search analysis (blastp using the nr database) can be used for molecular typing based on the VP4-pVP2 sequence. Regarding NCBI BLAST results, the default sorting parameter should be changed from the "E-value" (expectation value) to the "Per. Ident" (Percent Identity) column, as the E-value can be influenced by sequence length matching between the query and reference. Thus, adjusting this parameter is important for accurate typing. The notable exceptions to the 95% threshold were PV1-PV2 inter-type identity (Table 1) and CVA6 intra-type identity (Fig 3A); however, they were still discriminable in the blastp analysis. Despite a few limitations, this study shows that rapid diagnosis using VP4-pVP2 typing may facilitate routine clinical or environmental surveillance, as well as emergency outbreak responses. This study confirmed the reliability of VP4-pVP2-based typing and proposes a threshold for typing. This threshold can be applied in laboratory settings where EV testing is implemented.

## Supporting information

S1 Data Multi-FASTA file containing the VP4-pVP2 amino acid sequences of the 249 prototypes. (FASTA)

S1 Table Original table for pairwise identity comparisons of EVs and RVs in Fig 1B. (CSV)

S2 Table Summary of pairwise identity comparisons of EVs and RVs. (DOCX)
