# Supplementary material for: Evaluation of VP4-VP2 sequencing for molecular typing of human enteroviruses
Source: PLoS One. 2024 Dec 10;19(12):e0311806. doi: 10.1371/journal.pone.0311806 (PMC11630573; doi:10.1371/journal.pone.0311806)
Supplement: S2 Table — (DOCX) [file pone.0311806.s003.docx]

**Table S2 Summary for pairwise identity comparison of EVs and RVs.**

|  |  | EV-C | EV-B | RV-B | EV-A | EV-D | RV-A | RV-C |
| --- | --- | --- | --- | --- | --- | --- | --- | --- |
| EV-C | max | 96.62 |  |  |  |  |  |  |
|  | min | 75.00 |  |  |  |  |  |  |
|  | average | 82.73 |  |  |  |  |  |  |
| EV-B | max | 79.05 | 97.97 |  |  |  |  |  |
|  | min | 62.16 | 77.02 |  |  |  |  |  |
|  | average | 68.45 | 85.58 |  |  |  |  |  |
| RV-B | max | 75.67 | 70.27 | 100 |  |  |  |  |
|  | min | 60.13 | 56.75 | 82.43 |  |  |  |  |
|  | average | 67.70 | 61.70 | 90.57 |  |  |  |  |
| EV-A | max | 75.00 | 70.27 | 66.89 | 96.62 |  |  |  |
|  | min | 59.45 | 54.05 | 56.75 | 69.59 |  |  |  |
|  | average | 65.57 | 60.97 | 61.38 | 79.88 |  |  |  |
| EV-D | max | 66.21 | 64.86 | 59.45 | 72.97 | 93.24 |  |  |
|  | min | 59.45 | 55.40 | 56.75 | 56.75 | 90.54 |  |  |
|  | average | 63.09 | 59.48 | 58.09 | 65.40 | 91.89 |  |  |
| RV-A | max | 67.56 | 66.21 | 65.54 | 63.51 | 60.81 | 100 |  |
|  | min | 55.4 | 52.02 | 54.05 | 51.35 | 53.37 | 79.72 |  |
|  | average | 60.91 | 58.26 | 60.36 | 57.49 | 56.51 | 90.50 |  |
| RV-C | max | 66.21 | 64.86 | 62.83 | 62.83 | 61.48 | 66.89 | 100 |
|  | min | 55.40 | 52.02 | 54.05 | 52.70 | 56.08 | 55.40 | 75.67 |
|  | average | 60.86 | 57.66 | 58.36 | 57.73 | 58.87 | 61.79 | 82.71 |
